# Clinical, Virological and Immunological Subphenotypes in a Cohort of Early Treated HIV-Infected Children

**DOI:** 10.3389/fimmu.2022.875692

**Published:** 2022-05-03

**Authors:** Sara Domínguez-Rodríguez, Alfredo Tagarro, Caroline Foster, Paolo Palma, Nicola Cotugno, Sonia Zicari, Alessandra Ruggiero, Anita de Rossi, Annalisa Dalzini, Savita Pahwa, Stefano Rinaldi, Eleni Nastouli, Anne-Geneviève Marcelin, Karim Dorgham, Delphine Sauce, Kathleen Gartner, Paolo Rossi, Carlo Giaquinto, Pablo Rojo

**Affiliations:** ^1^ Pediatric Infectious Diseases Unit, Fundación para la Investigación Biomédica del Hospital 12 de Octubre, Madrid, Spain; ^2^ Department of Pediatrics, Fundación para la Investigación e Innovación Biomédica del Hospital Universitario Infanta Sofía y Hospital Universitario del Henares, Madrid, Spain; ^3^ Department of Pediatrics, Imperial College Healthcare National Health Service (NHS) Trust., London, United Kingdom; ^4^ Clinical and Research Unit of Clinical Immunology and Vaccinology, Academic Department of Pediatrics, Istituto di Ricovero e Cura a Carattere Scientifico (IRCCS) Ospedale Pediatrico Bambino Gesu, Rome, Italy; ^5^ Department of Systems Medicine, University of Rome “Tor Vergata”, Rome, Italy; ^6^ Multivisceral Transplant Unit, Department of Surgery, Oncology and Gastroenterology, Section of Oncology and Immunology, University of Padua, Padua, Italy; ^7^ Department of Microbiology and Immunology, University of Miami Miller School of Medicine, Miami, FL, United States; ^8^ Infection, Immunity & Inflammation Department, University College of London (UCL) Great Ormond Street Institute of Child Health (GOS), London, United Kingdom; ^9^ Sorbonne Université, INSERM, Institut Pierre Louis d’Epidémiologie et de Santé Publique, AP-HP, Centre d’Immunologie et des Maladies Infectieuses, Cimi-Paris, Paris, France; ^10^ Sorbonne Université, Inserm, Centre d’Immunologie et des Maladies Infectieuses, Cimi-Paris, Paris, France; ^11^ Academic Department of Pediatrics (DPUO), Istituto di Ricovero e Cura a Carattere Scientifico (IRCCS) Ospedale Pediatrico Bambino Gesu, Rome, Italy

**Keywords:** subphenotypes, immune signatures, viral dynamics, reservoir, perinatal, HIV, pediatric

## Abstract

**Background:**

Identifying subphenotypes within heterogeneous diseases may have an impact in terms of therapeutic options. In this study, we aim to assess different subphenotypes in children living with human immunodeficiency virus (HIV-1), according to the clinical, virological, and immunological characteristics.

**Methods:**

We collected clinical and sociodemographic data, baseline viral load (VL), CD4 and CD8 count and percentage, age at initiation of ART, HIV DNA reservoir size in peripheral blood mononuclear cells (PBMCs), cell-associated RNA (CA-RNA), ultrasensitive VL, CD4 subsets (T effector CD25+, activated memory cells, Treg cells), humoral-specific HIV response (T-bet B cells), innate response (CD56dim natural killer (NK) cells, NKp46+, perforin), exhaustion markers (PD-1, PD-L1, DNAM), CD8 senescence, and biomarkers for T-lymphocyte thymic output (TREC) and endothelial activation (VCAM). The most informative variables were selected using an unsupervised lasso-type penalty selection for sparse clustering. Hierarchical clustering was performed using Pearson correlation as the distance metric and WARD.D2 as the clustering method. Internal validation was applied to select the best number of clusters. To compare the characteristics among clusters, boxplot and Kruskal Wallis test were assessed.

**Results:**

Three subphenotypes were discovered (cluster1: n=18, 45%; cluster2: n=11, 27.5%; cluster3: n=11, 27.5%). Patients in cluster1 were treated earlier, had higher baseline %CD4, low HIV reservoir size, low western blot score, higher TREC values, and lower VCAM values than the patients in the other clusters. In contrast, cluster3 was the less favorable. Patients were treated later and presented poorer outcomes with lower %CD4, and higher reservoir size, along with a higher percentage of CD8 immunosenescent cells, lower TREC, higher VCAM cytokine, and a higher %CD4 PD-1. Cluster2 was intermediate. Patients were like those of cluster1, but had lower levels of t-bet expression and higher HIV DNA reservoir size.

**Conclusions:**

Three HIV pediatric subphenotypes with different virological and immunological features were identified. The most favorable cluster was characterized by a higher rate of immune reconstitution and a slower disease progression, and the less favorable with more senescence and high reservoir size. In the near future therapeutic interventions for a path of a cure might be guided or supported by the different subphenotypes.

## Introduction

The cure of human immunodeficiency virus (HIV-1) represents an unprecedented scientific challenge (1). Although antiretroviral therapy (ART) succeeded in reducing morbidity and mortality in HIV-1 infected individuals, it fails to clear HIV-1 infection. The resilience of the HIV-1 reservoir (2), together with the high mutation rate of the virus, the resistance to drugs (3), and the inability to generate neutralizing antibodies are major difficulties that must be overcome.

Soon after the primary infection, a reservoir of long-lived, resting memory CD4 cells containing integrated HIV-1 DNA is established. Since infants have a limited number of memory cells, compared to adults, early treated perinatally infected infants may pose the best chance of clearing the HIV-1 DNA and several strategies have been proposed to clear the virus on these patients. However, the success of vaccines or therapeutic approaches have been limited and very heterogeneous among patients (1, 4). Finding the reasons is a significant challenge. The answers might be lying in the great diversity of the virus and the variability of the host immune background of the patients. Significant effort has been made to identify the main factors associated with the HIV-1 reservoir size in children. Baseline viremia and replicative capacity (5), host immune background (6, 7), early treatment (8–10), and immune activation (11–13) have been associated with the HIV-1 reservoir size. However, each of these determinants are associated with small effects and individually do not effectively predict the reservoir size.

Multidisciplinary consortia have been created in the last years to study the reservoir and the complex immunological response to HIV. One of these consortia is the EPIICAL Project (Early treated Perinatally HIV-infected Individuals: Improving Children’s Actual Life with Novel Immunotherapeutic Strategies), which aims to establish a predictive *in vitro* and *in vivo* platform to inform treatment strategies leading to HIV remission. Usually, in pediatric HIV, these consortia studies provide high-dimensional information from small sample sizes. Numerous databases are created and populated as a result. The benefits and challenges of accumulating large amounts of biological information and modeling complex variable interactions are more tangible than ever. Integrating multi-level information could reduce the prediction model’s complexity and provide a unique opportunity to identify clinically relevant HIV-1 subphenotypes.

As in other disease areas (14–16), in the HIV area there is an urgent need to reveal subphenotypes integrating immune, virologic and clinical information, that can lead to personalized HIV-1 medicine. Distinct phenotypes may respond differently to different therapies in proof-of-concept trials. In the future, we may be able to design directed, individualized therapeutic approaches for HIV-1 infected children pursuing a path to a cure.

In this study, we aim to identify and characterize pediatric subphenotypes according to clinical, virologic, and immunologic features of patients in a cohort of children and adolescents with early-treated perinatally-acquired HIV-1.

## Methods

### Study Population

Child and Adolescent Reservoir Measurements on early suppressive ART (CARMA) Study (17) was a multicenter, cross-sectional study enrolling subjects with HIV from 7 European centers contributing to EPIICAL; in England, Spain and Italy. A total of 40 perinatally HIV-1 infected children were enrolled. Eligibility criteria were i) initiating ART within the first 24 months of life, ii) sustained viral suppression, defined as a viral load (VL) of less than 400 copies/mL achieved within 12 months of ART initiation and maintained for a minimum of 5 years with a viral load of <50 copies/mL. Children with concurrent viral hepatitis or TB coinfection, malignancies, or concomitant immunosuppressive therapy were excluded. Data collection included demographics, age at HIV diagnosis, age at initiation of ART, age at the time of analysis, and ART drug regimens ever received. Immunology variables (CD4 total count, CD8 total count, corresponding percentages, CD4:CD8 ratio) and VL were recorded at diagnosis, ART initiation, throughout treatment, and at time of viral reservoir analysis. Baseline was defined as the first measurement recorded for the patient before or at ART initiation. A viral load blip was defined as a rise in plasma VL from 50 to 399 copies/mL returning to <50 copies/mL on repeat sampling.

### Laboratory Analysis

The CARMA study main endpoints has been previously described, including the total HIV-1 DNA quantification per 10^6^ peripheral blood mononuclear cells (PBMC) by quantitative polymerase chain reaction (qPCR) (17). Human immunodeficiency virus Western blot (WB) kit 2.2 (Medical Systems, Genova, Italy) was used, following the manufacturer’s instructions, to detect specific antibody (Ab) responses, in 20 μL of plasma, against 10 different HIV-1 viral proteins (gp160, gp120, p66, p55, p51, gp41, p39, p31, p24, and p17) as previously described. A WB score value was calculated according to the bands intensity score (18, 19). HIV-1 DNA copies on CD4 cells, relative telomere length (as a marker of cellular senescence) and levels of T-cell receptor rearrangement excision (TREC) by PCR. Immunological profile was assessed by flow cytometry as described in Dalzini A et al. (20).

The natural killer (NK) phenotype and function were analyzed by flow cytometry and transcriptional profile of PBMCs by RNA-Seq (21). The single molecule array (SIMOA) assay was performed for HIV-1 p24 and some cytokines (PD-L1, IL-10, IL-6, TNF-α, PD-1) whereas levels of IP-10, MCP-1, vascular cell adhesion molecule-1 (VCAM-1) were assessed by Procartaplex using ultrasensitive Luminex technology according to the manufacturers protocols (Quanterix and Invitrogen, respectively).

The B-cell phenotype and intracellular T-bet expression profile (MFI) was measured by flow Cytometry. After washing with PBS 10% FBS, cells underwent surface staining with the following monoclonal antibodies (mAbs, from BD Biosciences): CD3, CD10, CD16 (BV510), CD19 (APC-R700), CD21 (APC), CD27 (FITC), IgD (BV421), IgM (PE-CF594), IgG (BV605), CD11C (PC-7). Finally, stained cells were acquired on Cytoflex (Beckman Coulter, Brea, CA) and analyzed with FlowJo v10.0.8 (Tree Star) software. Following surface staining fixing and permeabilization of cells (BD permeabilization solution II 1x), cells were stained with an anti T-bet BV650 (04-46, BD). For T-cell phenotype, LIVE/DEAD Fixable Blue Dead Cell Stain Kit from Thermo Fisher Scientific (Boston, MA) was used to detect and exclude dead cells. After washing with PBS 10% FBS, cells underwent surface staining with the following monoclonal antibodies as previously described (22). Gating strategies for B-cell phenotypes, T-bet and CD11c are provided in Ruggiero A et al. (23). Gating strategies for T-cell are shown in Rinaldi S. et al. (22). Positive cell gating was set using fluorescence minus one control. All the reagents were tested and titrated for optimum concentration before usage.

### Statistical Analysis

Study population characteristics were described using medians and interquartile ranges (IQR) and absolute numbers with frequencies for categorical variables.

To analyze and describe the subphenotypes within the study population, we first selected the most informative features from a large set of features (*p*=60), by using an unsupervised lasso-type penalty selection method for sparse clustering implemented in the sparcl R package (24). We selected the most important features by a sequential forward search (SFS). This SFS starts with zero features and sequentially adds one feature at a time starting with the highest feature weights. The inclusion of features stops when adding more features does not improve the maximum likelihood criterion according to mclust R package (25). A total of 25 variables with the highest feature weights were selected.

Subphenotypes were determined using normalized values of each variable by unsupervised hierarchical clustering analyses using Pearson correlation distance and Ward.D2 algorithm as the distance metric. Data processing and clustering analysis were performed using the pheatmap R package (26). Internal cluster validation was performed to determine the optimal number of clusters with the NbClust R package (27). Chi-squared and Fisher tests were applied to assess differences among the categorical variables of the different subphenotypes. For continuous variables, Kruskal-Wallis tests were applied. Missing data were completed through a non-parametric random forest imputation technique for mixed-type data. This method was implemented by using the Missforest R package (28). The normalized root mean squared error (NRMSE) and the proportion of falsely classified (PFC) were assessed.

## Results

### Study Population

The socio-demographic, clinical, virological, and immunological features for the 40 perinatally HIV-1 infected children are described in **Table 1**. Most of the children were female (68%), and a total of 32.5% were of black African origin. They were diagnosed with HIV-1 at a median of 4 months of age (IQR: 2.19-6.32) and initiated ART at a median of 4 months of age as well (IQR: 2.19-6.23). At baseline, children had a median CD4 count of 1515 cells/mm^3^ (IQR:637-2235), median CD4 percentage of 31.0% (IQR:18.0-38.0), and a median log10 VL of 5.28 copies/mL (IQR: 4.07-5.70). Immunological and virological analyses of the samples were done at a median of 12 years (IQR: 8.0-15.6) after ART initiation. The total HIV-1 DNA had a median value of 48.3 (IQR: 6.65-113) copies/10^6^ PBMC and 255 (IQR: 75.0-434) on CD4 cells. The variables included as potential determining factors in the subphenotypes characterization are summarized according to the percentage of missing information in (**Supplementary Figure 2**). The NRMSE value obtained in the imputation was 0.022 and the PFC was 0.07. The imputed dataset was compared with the complete-cases dataset. Results are shown in **Supplementary Table 1**. No significant difference was observed between the complete-cases database and the imputed database.

**Table 1:** Study population characteristics.

	[ALL]	N
	* N = 40 *	
**Gender:**		40
Male	13 (32.5%)	
Female	27 (67.5%)	
**Age**		
At HIV-1 diagnosis (months)	4.17 [2.19;6.32]	40
At ART (months)	4.08 [2.19;6.23]	40
At HIV-1 DNA reservoir measurement (years)	12.2 [8.03;15.6]	40
**ART regimen at initiation:**	** **	40
Triple NRTI	2 (5.00%)	
NNRTI	1 (2.50%)	
NRTI	1 (2.50%)	
NRTI + NNRTI	23 (57.5%)	
NRTI + PI	12 (30.0%)	
PI	1 (2.50%)	
**At Baseline**		
HIV-1 RNA viral load (copies/mL) (log10)	5.28 [4.07;5.70]	40
% CD4^+^ cells	31.0 [18.0;38.0]	33
CD4 total count	1515 [637;2235]	29
% CD8 ^+^ cells	32.0 [25.0;40.0]	29
**Time to suppression (months)**	4.69 [2.52;6.26]	40
**Anti-CMV IgG:**		39
Negative	10 (25.6%)	
Positive	29 (74.4%)	
**Anti-CMV IgM**	5.21 [5.00;7.47]	38
Negative	38 (100%)	
**Antigen/Antibody relation 4th generation Abbot:**		39
Equivocal	2 (5.13%)	
Non Reactive	10 (25.6%)	
Reactive	27 (69.2%)	
**Western blot**		39
Western blot score	1.00 [0.50;2.00]	39
**Virologic features**		
HIV DNA reservoir (DNA per 10^6^ copies/mL PBMC)	48.3 [6.65;113]	40
HIV DNA reservoir (CD4^+^ cells/mm^3^)	255 [75.0;434]	35
HIV Cell-Associated RNA LTR (10^6^ copies/mL PBMC)	2.73 [0.00;44.1]	40
HIV Cell-Associated RNA pol (10^6^ copies/mL PBMC)	0.00 [0.00;1.38]	40
Last ultrasensitive viral load measurement (copies/mL)	2.37 [1.05;2.72]	40
**NK cells subpopulations**		
%NK PBL	5.03 [2.39;6.72]	38
%CD56dim	75.0 [68.8;81.5]	38
%CD56-	9.34 [5.57;16.7]	38
%NKp46+	60.7 [49.9;74.0]	23
%DNAM-1+	83.9 [77.2;89.4]	23
CD107 Not-Stimulated	6.41 [4.37;11.4]	35
**Immunological profile**		
% CD4^+^ CD28– CD57+ immunosenescent cells	1.63 [0.57;2.90]	35
% CD8^+^ CD28– CD57+ immunosenescent cells	12.7 [8.01;16.8]	35
% Activated cells CD4^+^ cells	0.37 [0.26;0.54]	35
% Activated cells CD8^+^ CD38+ HLADR+ cells	1.55 [1.00;2.00]	35
Relative Telomere length CD4^+^ cells	1.33 [1.22;1.57]	36
Relative Telomere length CD8^+^ cells	1.40 [1.25;1.52]	35
TREC (PBMC)	1720 [846;2730]	37
% CD4 Effector CD38- HLA-DR+	1.73 [1.09;2.41]	33
% CD4 CD45RO+ CD27+ TTM ICOS+	11.0 [7.56;17.2]	36
% CD4 CD45RO+ CD27+ TTM Q10 CD38+ HLA-DR+	0.49 [0.30;0.66]	35
% CD4 Effector CD25	24.6 [18.5;28.4]	36
% CD4 Effector TIGIT	1.62 [1.25;2.08]	36
% CD4 TIGIT	2.67 [2.26;3.63]	36
% CD8 Naive TIGIT	2.99 [2.29;3.74]	36
% CD4 CD40L	0.74 [0.44;1.98]	34
% CD4 PD-1	4.12 [3.03;6.70]	36
**B-cells T-bet expression**		
Naïve	188 [157;224]	40
Activated Memory	248 [204;325]	40
Double Negative IgD- CD27-	197 [143;237]	40
Resting memory IgD- IgM- IgG+	186 [133;224]	40
CD19+ CD10- IgD- IgG- IgM+	205 [154;256]	40
**Cytokines**		
PDL-1 (pg/mL)	63.4 [53.4;76.4]	40
IL-10 (pg/mL)	1.37 [1.03;1.66]	40
IL-6 (pg/mL)	0.88 [0.66;1.28]	40
TNF-α (pg/mL)	3.04 [2.33;3.74]	40
PD-1 (pg/mL)	389 [252;576]	40
IP-10 (pg/mL)	2.89 [1.98;4.64]	40
MCP-1 (pg/mL)	3.97 [2.64;4.82]	39
VCAM (pg/mL)	28842 [16731;42854]	26

ART, Antiretroviral treatment; NRTI, Nucleoside reverse-transcriptase inhibitors; NNRTI, Non-nucleoside reverse-transcriptase inhibitors; PI, Protease Inhibitors; CMV, Cytomegalovirus; Ig, immunoglobulin; PBMC, peripheral blood mononuclear cell; PBL, peripheral blood lymphocyte; Ag, antigen; Ab, antibody.

### Features Selection

The importance weights for the remaining features are plotted in **Supplementary Figure 3** According to the unsupervised sparse feature selection, the features most responsible for the differences between patients were, in order of importance, baseline viral load, VCAM cytokines, levels of T-cell receptor excision circles (TREC), cell-associated HIV-1 RNA (LTR), HIV-1 reservoir size on CD4 cells, PD-1, total HIV Reservoir size (PBMC), T-bet expression on AM, CD19+ CD10-IgD-IgG-IgM+, RM Moir IgD-IgM+, RM IgD- IgM-IgG+, IgD-CD27-, and naïve, cell-associated RNA (pol), % CD4 Effector CD25, PDL-1, %CD56dim, %CD4 Q2 CD45RO+ CD27+ TTM ICOS+, Baseline % CD4, % NKp46, ultrasensitive viral load, immunosenescent cells %CD8, baseline %CD8, % DNAM-1+, %Perforin+, and age at ART.

### Three Different Clusters Were Defined

**Figure 1:**
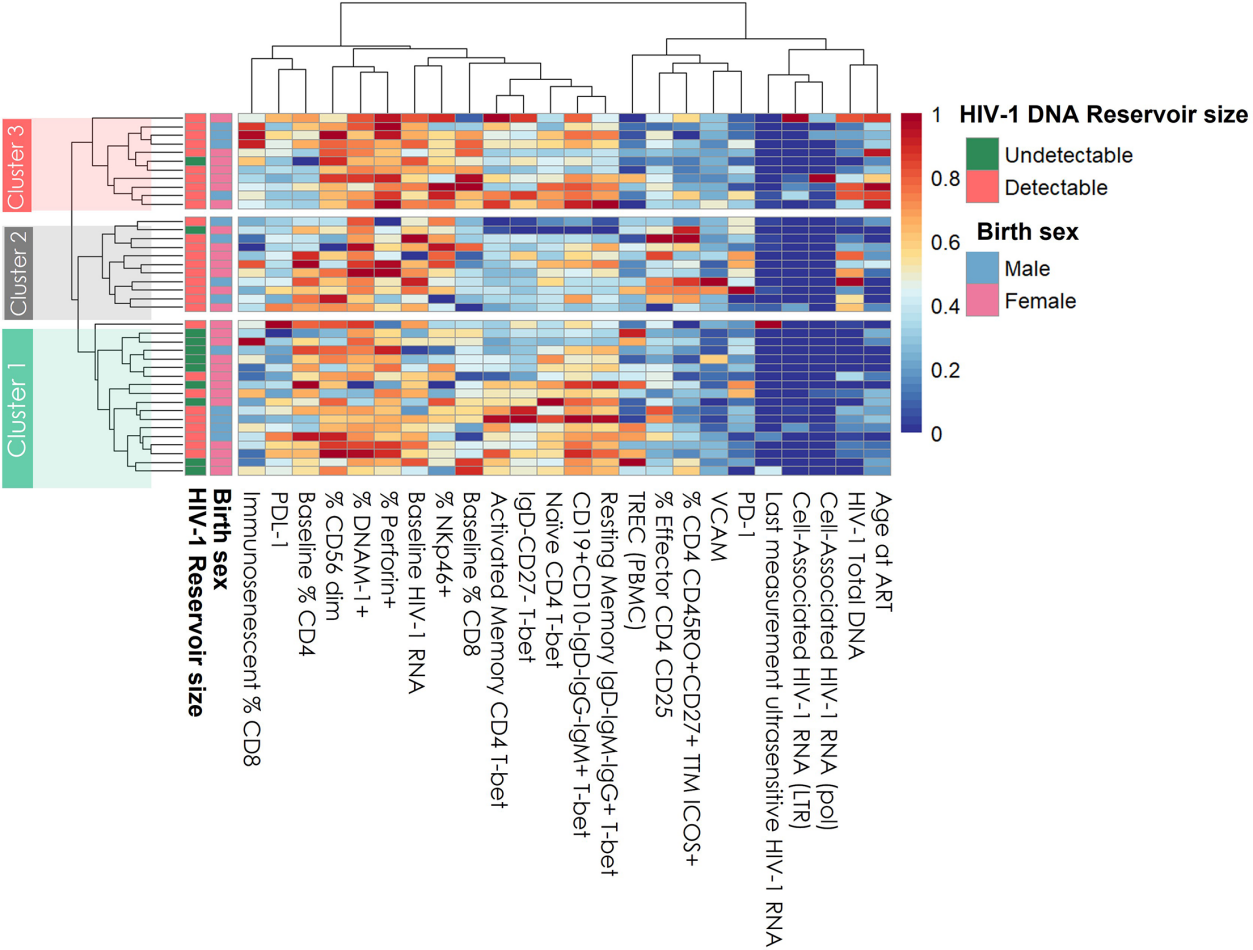
Hierarchical Clustering of all patients based on the clinical, immunological, and virological features. Columns on heatmap represent patient features. Rows are each of the subjects in the study (n = 40). Normalized values are plotted: red color means higher values; blue color means lower values. ART, antiretroviral therapy; VL, viral load; TREC, T-cell receptor excision circle; Undetectable, ≤10copies/mL; Detectable >10 copies/mL.

Three statistically robust clusters were defined within the study population (**Figure 1**). The cluster 1 showed clinical, immunological and virological features in general favorable; the cluster 3 showed values in general less favorable, and cluster 2 showed intermediate features.

The heatmap shown in **Figure 1** represents how patients (rows) are aggregated according to the features (columns) in the three clusters: cluster 1 (n=18), cluster 2 (n=11), and cluster 3 (n=11). Low values were colored in blueish and high values in reddish colors. There were no significant differences in gender across the clusters with 72.2% of females in cluster 1, 63.6% in cluster 2, and 63.6% in cluster 3 (p=0.847). Likewise, no significant differences were found in age at sample extraction among clusters (12.7 years [9.67-14.9] vs. 8.7 years [6.3-13.0] vs 15.4 years [8.7-16.5], p=0.207).

There were more patients with undetectable DNA reservoir (<10 copies/mL 10^6^ PBMC) in cluster 1 (50%) than in cluster 2 (9.1%) and cluster 3 (9.1%) (p=0.016).

**Figure 2:**
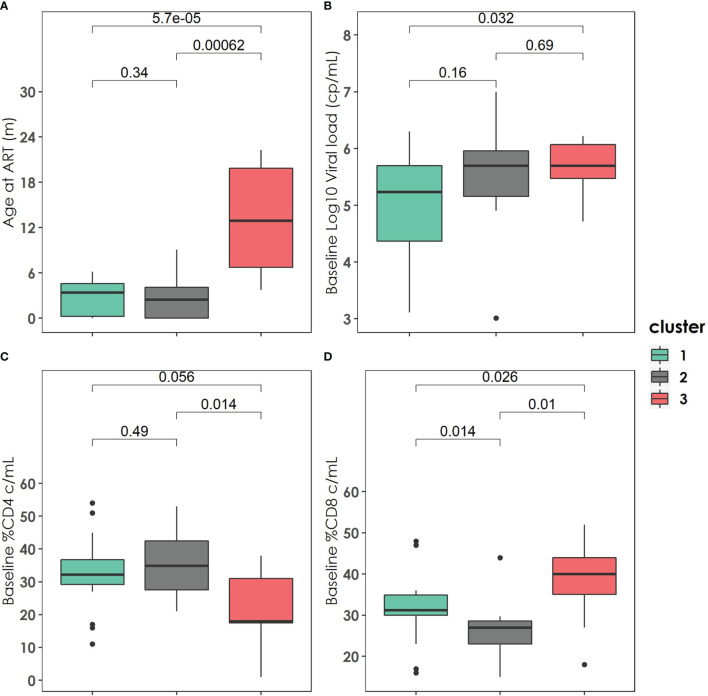
Baseline clinical, immunological, and virological characteristics of the three clusters described. **(A)** Age at antiretroviral treatment distribution among the three clusters. **(B)** Baseline Log10 Viral load (copies/mL) distribution among the three clusters. **(C)** Baseline % CD4 (cell/mL) distribution among the three clusters. **(D)** Baseline %CD8 (cell/mL) distribution among the three clusters. P-values were calculated using U-Mann Whitney Test and Kruskal-Wallis test when appropriate.

The patients in cluster 3 started ART later than patients in cluster 2 (12.9 months [6.74-19.87] *vs.* 2.46 months [0-4.08], p=0.0062) or cluster 1 (12.9 months [6.74-19.87] *vs.* 3.4m [0.2-4.58], p=5.7 ·10^-05^). In addition, cluster 3 had higher viral loads, higher baseline CD8 percentages, and lower CD4 percentages. Interestingly, the viral load measured at the end of follow-up was significantly higher in cluster 2 than in cluster 1 (**Figure 2**).

**Figure 3:**
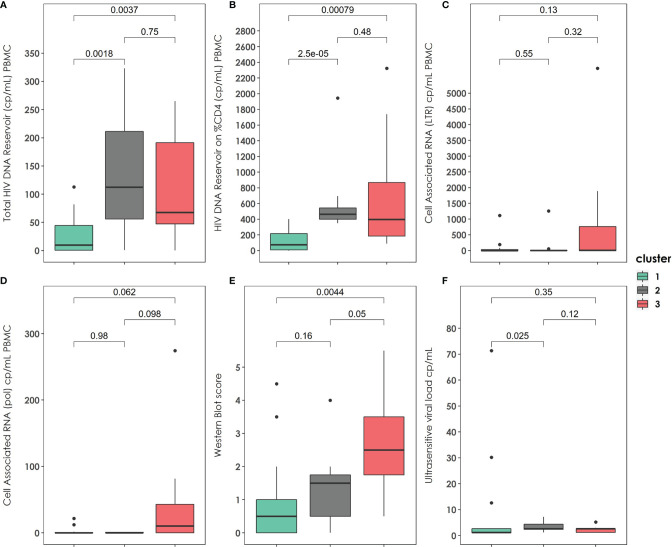
Virological characteristics and Western-Blot score of the three clusters described at the end of the follow up. **(A)** Total HIV reservoir size (copies/mL) on PBMC distribution among the clusters. **(B)** HIV DNA reservoir size on %CD4 distribution among the clusters. **(C)** Cell Associated RNA at LTR region (copies/mL) distribution among the clusters. **(D)** Cell Associated RNA at pol region (copies/mL distribution among the clusters). **(E)** Western blot score distribution among the clusters. **(F)** Ultrasensitive viral load measurement distribution among the clusters. PBMC: peripheral blood mononuclear cell. P-values were calculated using U-Mann Whitney Test and Kruskal-Wallis test when appropriate.

Moreover, the patients in cluster 1 had significantly lower HIV-1 DNA reservoir size (PBMC) than patients in cluster 2 (9.65 [0.47-44.56] *vs.* 112.4 [55.75-211.4], p=0.002) or cluster 3 (9.65 [0.47-44.56] *vs.* 67.7 [47.2-191.2], p=0.004). No differences in total HIV DNA reservoir (PBMC) were found between patients in clusters 2 and 3. Likewise, patients in cluster 1 had significantly lower HIV DNA reservoir in CD4 cells (**Figures 3A, B**). There were no significant differences found among clusters according to cell-associated HIV-1 RNA either in LTR or pol. However, there is a suggestive trend of higher cell-associated HIV-1 RNA values in patients of cluster 3 (**Figures 3C, D**). In **Figure 3E** we can observe a western blot score value growing trend among the three clusters, being the patients in cluster 3 those with the highest values (0.5 [0-1.0] *vs.* 1.5 [0.5-1.75] *vs.* 2.5 [1.75-3.5]). The patients in cluster 1 presented significantly lower viral load measured with ultrasensitive techniques than those in cluster 2 (**Figure 3F**).

**Figure 4:**
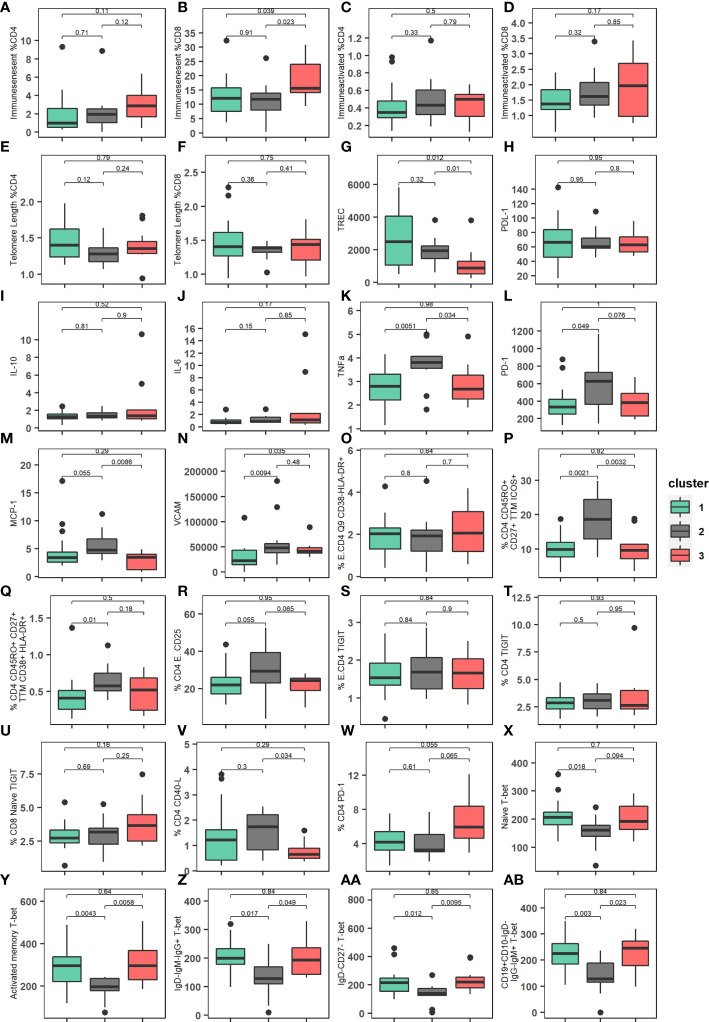
Immunological characteristics of the three clusters described. **(A)** Percentage of immunosenescent CD4 cells among the clusters. **(B)** Percentage of immunosenescent CD8 cells among the clusters. **(C)** Percentage of immunoactivated CD4 cells among the clusters. **(D)** Percentage of immunoactivated CD8 cells among the clusters. **(E)** Relative Telomere length of percentage of CD4 cells among the clusters. **(F)** Relative Telomere relative length of percentage of CD8 cells among the clusters. **(G)** TREC (T-cell receptor rearrangement excision circle) levels/10^5^ PBMC among the clusters. **(H)** PDL-1 expression among the clusters. **(I)** IL-10 (pg/mL) expression among the clusters. **(J)** IL-6 (pg/mL) expression among the clusters. **(K)** TFN-alpha (pg/mL) expression among the clusters. **(L)** PD-1expression among the clusters. **(M)** MCP-1 (pg/mL) expression among the clusters. **(N)** VCAM (pg/mL) expression among the clusters. **(O)** Percentage of Effector CD4 T cell expression CD38- HLA-DR+ among the clusters. **(P)** Percentage of CD4 expression CD45RO+ CD27+ TTM ICOS+ among the clusters. **(Q)** Percentage of CD4 CD45RO+ CD27+ TTM CD38+ HLA-DR+ among the clusters. **(R)** Percentage of Effector CD4 CD25+ among the clusters. **(S)** Percentage of Effector CD4 TIGIT receptor among the clusters. **(T)** Percentage of CD4 TIGIT receptor among the clusters. **(U)** Percentage of CD8 Naïve TIGIT receptor among the clusters. **(V)** Percentage of CD4 CD40-L among the clusters. **(W)** Percentage of CD4 PD-1 among the clusters. **(X)** Distribution of Naïve CD4 T-bet expression among the clusters. **(Y)** Distribution of Activated memory CD4 T-bet expression among the clusters. **(Z)** Distribution of Resting memory IgD- IgM- IgG- T-bet expression among the clusters. **(AA)** Distribution of IgD- CD27- T-bet expression among the clusters. **(AB)** Distribution of CD19+CD10-IgD-IgG-IgM+ T-bet expression among the clusters. P-values were calculated using Kruskal-Wallis test.

The patients in cluster 3 presented a higher percentage of CD8 immunosenescent cells, a lower TREC values, a higher VCAM cytokine, and a higher percentage of CD4 PD-1. The patients in cluster 2 presented lower T-bet expression on Naïve Moir, AM Moir, RM Moir, and Double negative cells, a higher percentage of CD4 effector cells, higher % CD4 Q2 CD45RP+ CD27+ TTM ICOS+, and higher TNF-alpha (**Figure 4**).

**Figure 5:**
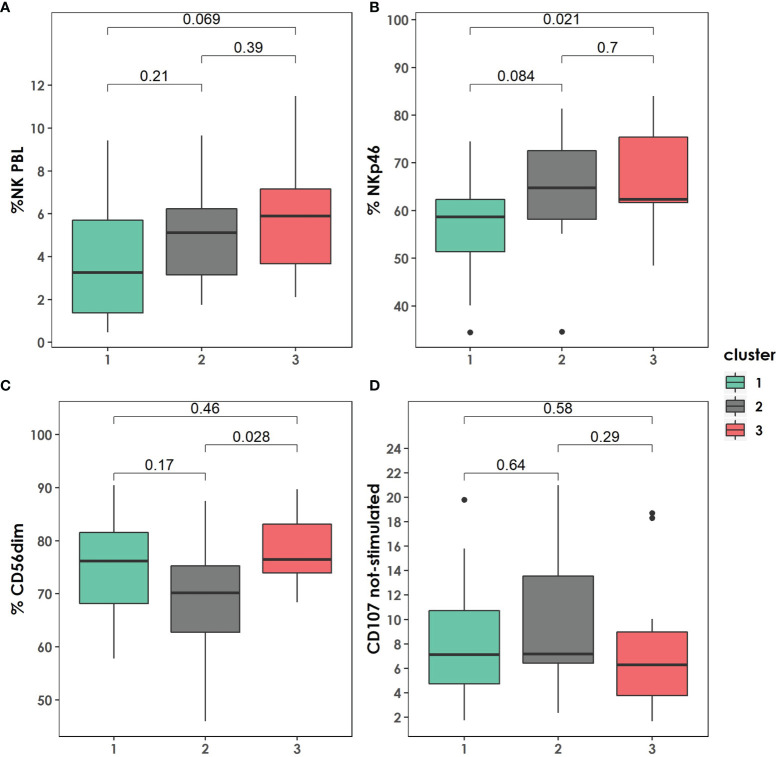
Distribution of Natural Killer (NK) subpopulations within the clusters described. **(A)** Total percentage of NK on peripheral blood lymphocytes distribution among the clusters. **(B)** Percentage of NKp46 distribution among the clusters. **(C)** Percentage of CD56dim distribution among the clusters. **(D)** CD107 not-stimulated distribution among the clusters. P-values were calculated using U-Mann Whitney Test and Kruskal-Wallis test when appropriate.

We observed a non-significant increasing trend of the percentage of NK cells on PBMC among clusters 1, cluster 2, and cluster 3. Likewise, patients in cluster 3 presented higher values of % NKp46, a difference only significant when comparing cluster 3 and cluster 1 (**Figure 5**).

According to virological and immunological follow-up, there were not statistically significant differences between the clusters according to the presence of blips (cluster 1: 3/18 (16.7%) vs. cluster 2: 5/11 (45.5%) vs. cluster 3: 3/11 (27.3%), p=0.269). Similarly, the patients in the different clusters presented similar median CD4 cell counts at the end of follow up [cluster 1: 788.7 (IQR, 555.8-1053.7) *vs.* cluster 2: 892.0 (IQR, 720.7-980.5) *vs.* cluster 3: 866.7 (IQR, 752.0-1096.9), p=0.835].

## Discussion

In a pediatric cohort with early-treated patients with long-term HIV viral control, three clusters were identified, revealing three distinct subphenotypes with specific clinical, virological, and immunological features. These three clusters were found in a pediatric cohort with a long-term viral control.

The most informative features selected to build the clusters were mainly virological. The baseline viral load was the feature with the highest percentage of importance according to this model, meaning that this feature is the one than better differentiate between patients. This results are in concordance of research previously published suggesting that HIV-1 RNA plasma levels are a better progression marker than immunological markers such as CD4 cell count (29).

The subphenotype composed by patients in cluster 1 is characterized by early (<3 months) treated patients, lower values of HIV-1 reservoir size at a median of 12 years (IQR: 7.3-15.4) after ART initiation, either in total PBMC DNA or on isolated CD4 cells, and low values of WB score which is an indicator of HIV-1-specific antibody concentration, as a proxy of low viral persistence. This subphenotype, with an apparent better immune and viral profile, was also characterized by higher values of TREC and lower values of VCAM, biomarkers for thymic output (30) and endothelial activation (31) respectively. In other words, this subphenotype is composed of patients with a higher rate of immune reconstitution and a slower disease progression 12 years after ART initiation. This finding is in agreement with previous studies performed in children that correlated TREC with the number of CD4 T cells in children (30, 32).

These biomarkers may be useful for screening of people living with HIV for therapeutical interventions directed to erase the reservoir.

In contrast, the subphenotype composed by patients in cluster 3 is characterized by later (>12 months) ART initiation, a lower baseline percentage of CD4 cells, a higher percentage of CD8 cells, and higher WB scores. We can observe that, although not statistically significant, this subphenotype has higher values of cell associated HIV-1 RNA. This ‘poor outcome’ subphenotype was also characterized by a higher percentage of immunosenescent CD8 cells, lower TREC values, a higher percentage of CD4 PD-1, and a higher percentage of NK cells. Thus, this subphenotype is associated with a poorer profile than cluster 1, higher cytotoxic response, and senescence. As in cancer, CD8+ T and the upregulation of PD-1 on CD8+ T cells may play a critical role in future immunotherapies (33). Concerning cell aging, this finding is in agreement with the fact that highly differentiated mature NK cells accumulate with aging, with decreasing ability to kill virus-infected target cells (34). In the future, this subset of patients may benefit from specific or more aggressive treatments that attenuate the activation of the immune system.

Interestingly, another subphenotype, patients in cluster 2, was identified. This phenotype was not only an unspecific subphenotype composed by patients unclassified in cluster 1 or cluster 3 but presented specific features that may indicate a signature. This subphenotype was characterized by early treated patients with a lower percentage of CD8 cells, lower TNF-alpha, higher PD-1, higher CD45RO+CD27+ TTM ICOS+, higher percentage of CD4 effector CD25+, and lower T-bet expression. In summary, this subphenotype was characterized by a weak innate and humoral immune response despite early treatment and a good baseline CD4 profile. There were statistically significant differences in the HIV-1 RNA viral load at the end of follow-up between cluster 1 and cluster 2, and more patients with blips during the follow up in cluster 2. This might be explained by a weak adherence in this subphenotype, but the difference is unlikely to be significant as all the patients were long-term suppressed. However, as a limitation of the study, we do not have specific ART adherence information.

These findings suggest that although these patients are early treated and have higher CD4% at baseline, their weak innate and adaptive responses drive them to high reservoir size and probably poorer outcomes than those in cluster 1. This finding supports the idea that treating early, and prolonged suppression is not the only factor involved in long-term reservoir size. Likely, genetic factors involving host response to the virus may play a role. Again, identifying these patients early may be useful for personalized medicine. They might benefit for therapies that enhance the immune response, as broadly neutralizing antibodies.

As the main limitation of this study, the sample size prevented us from externally validating the subphenotypes. However, a previous study showed that samples sizes of at least 40 subjects resulted in good (80% or higher) accuracy to detect the true number of clusters for a maximum of four separations (35). We believe that the strong methodology applied in this study and the robust internal validation performed to make the results valid. The widespread use of high-throughput technologies results in a rapid accumulation of complex data sets with a high number of predictors. Integrative clustering methods can lead to the discovery of novel disease signatures and deliver meaningful information for tailored therapies and precision medicine. However, larger studies will be needed to externally validate these subphenotypes.

In conclusion, within a cohort of early treated children with perinatally acquired HIV who were suppressed for years and might be considered clinically similar, we have found three distinct subphenotypes. The most favorable cluster was characterized by with a higher rate of immune reconstitution and a slower disease progression, and the less favorable by a higher CD8+ response, more senescence and high reservoir size. An intermediate group of patients in cluster 2 with apparently good baseline features performed poorly and could be helped with immune-enhancing therapies such as anti-PD1 immunotherapy. On the other hand, patients with the most favorable phenotype may be susceptible to be treated with block-and-lock strategies by silencing HIV-1 expression for a prolonged drug-free remission.

New therapeutic strategies could be tailored for these subphenotypes following the rapidly growing approach of precision medicine.

## Data Availability Statement

The data analyzed in this study is subject to the following licenses/restrictions: The informed consent of the patients did not include a statement to make public their pseudonymized data. Requests to access these datasets should be directed to caroline.foster5@nhs.net.

## Ethics Statement

The CARMA study was approved by ethics committees within each country and informed assent/consent obtained from adolescents and/or parent(s)/legal guardian in accordance with country-specific law. Each participant received a unique study number, under which data was pseudo-anonymised. Written informed consent to participate in this study was provided by the participants’ legal guardian/next of kin.

## Author Contributions

SD-R conceived the study and performed the statistical analysis. PRoj and AT designed and supervised the work. conceived, PP, NC, SZ, AR, ADR, AD, SP, SR, EN, A-GM, KD, DS, and KG planned and performed the laboratory experiments. PRoj and CG provided resources and supervised the study. All the authors provided critical feedback and helped shape the research, analysis, and manuscript. All authors contributed to the article and approved the submitted version.

## Funding

This work has been supported within EPIICAL project by through an independent ViiV Healthcare grant to the PENTA (Paediatric European Network for Treatment of AIDS) Foundation. The funders had no role in study design, data collection, analysis, and interpretation, or manuscript preparation. CARMA was supported by EPIICAL (Early-treated Perinatally HIV-infected Individuals: Improving Children’s Actual Life with Novel Immunotherapeutic Strategies) project, funded through an independent grant by ViiV Healthcare United Kingdom. This work is part of the EPIICAL project (http://www.epiical.org/), supported by PENTA-ID foundation (http://penta-id.org/).

## Conflict of Interest

The authors declare that the research was conducted in the absence of any commercial or financial relationships that could be construed as a potential conflict of interest.

## Publisher’s Note

All claims expressed in this article are solely those of the authors and do not necessarily represent those of their affiliated organizations, or those of the publisher, the editors and the reviewers. Any product that may be evaluated in this article, or claim that may be made by its manufacturer, is not guaranteed or endorsed by the publisher.

## Supplementary Material

Supplementary Figure 1 Gating strategies for B-cell phenotypes. Gating strategy of the B-cell phenotypes, T-bet, and CD11c are provided in the figure.

Supplementary Figure 2 Variable missing values. The percentage of missingness is plotted in x-axis for each of the cell subsets in y-axis. Variables such as IFNγ, %perforin +, and %NKp46+, or %DNAM-1+ present >15% of the information missing. In other words, less than 34/40 patients present a value of IFNγ, %perforin +, and %NKp46+, or %DNAM-1+ expression.

Supplementary Figure 3 Variable importance. Variable importance according to an unsupervised lasso-type penalty selection method for sparse clustering. We selected the most important features by a sequential forward search (SFS). A total of 25 variables with the highest feature weights were selected.

Supplementary Table 1 Differences between complete cases database and after imputation.
