# Supplementary figures and images for: Clinical, Virological and Immunological Subphenotypes in a Cohort of Early Treated HIV-Infected Children
Source: Front Immunol. 2022 May 3;13:875692. doi: 10.3389/fimmu.2022.875692 (PMC9111748; doi:10.3389/fimmu.2022.875692)

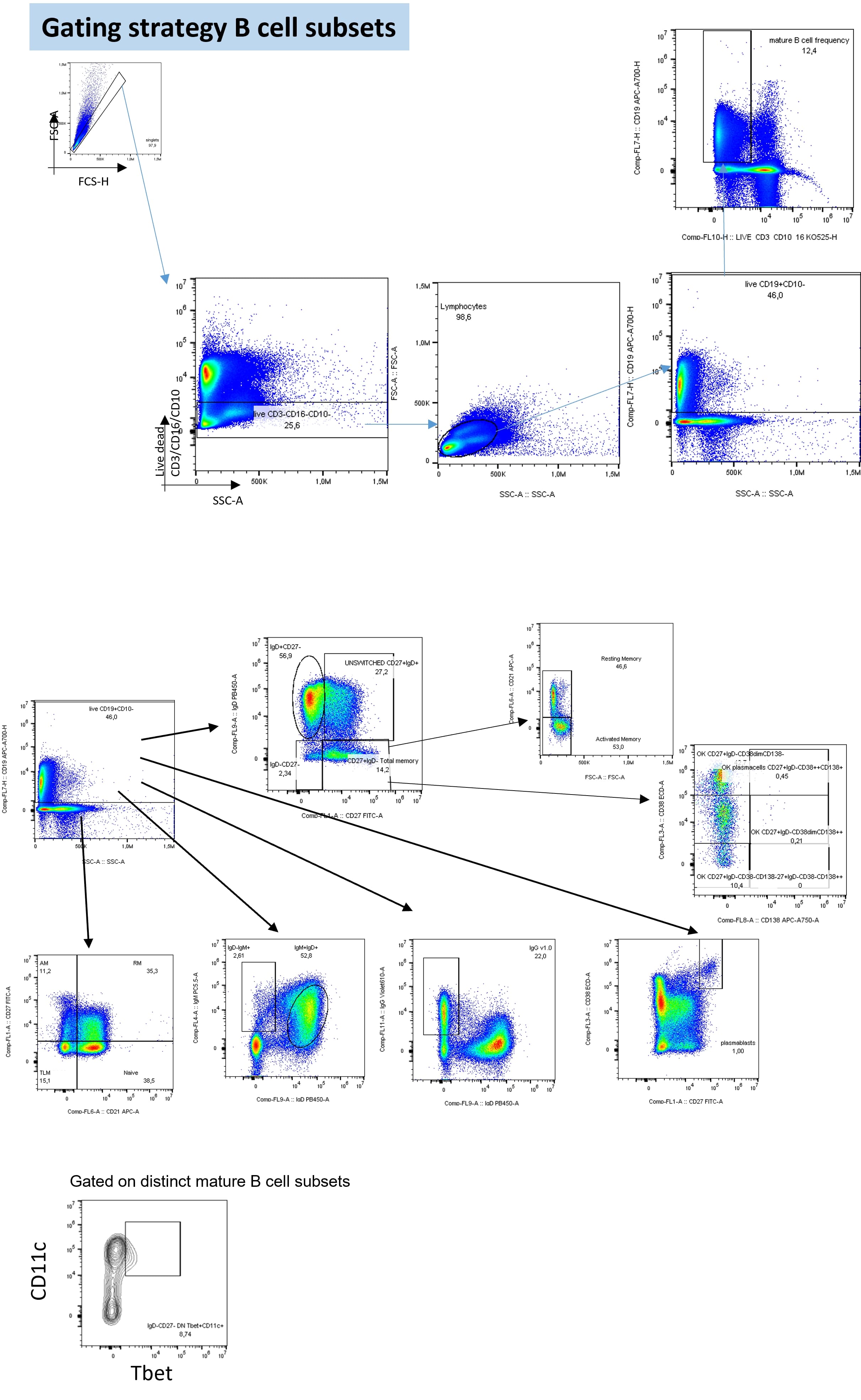

Supplement: Supplementary Figure 1 — Gating strategies for B-cell phenotypes. Gating strategy of the B-cell phenotypes, T-bet, and CD11c are provided in the figure. [file Image_1.jpeg]

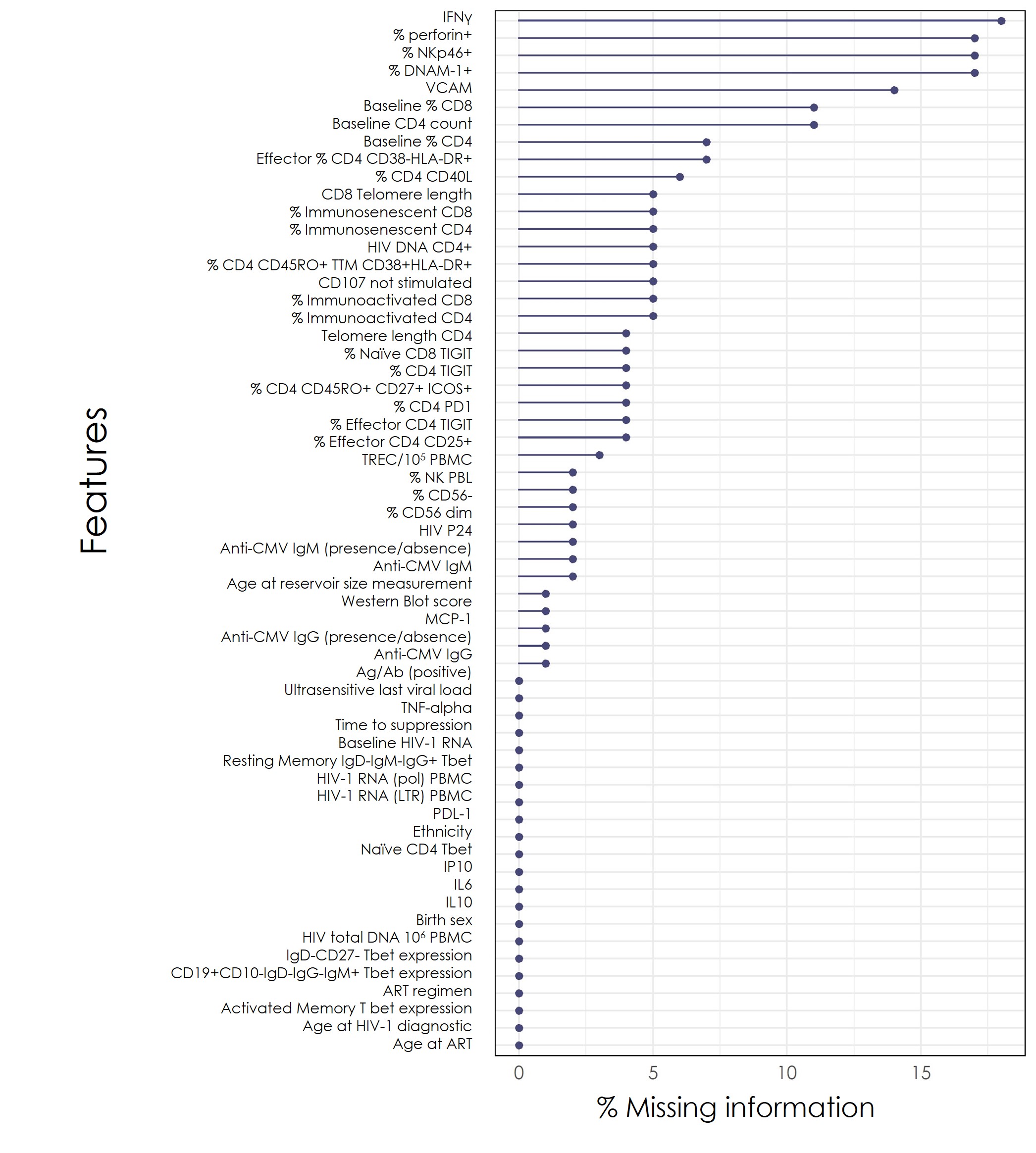

Supplement: Supplementary Figure 2 — Variable missing values. The percentage of missingness is plotted in x-axis for each of the cell subsets in y-axis. Variables such as IFNγ, %perforin +, and %NKp46+, or %DNAM-1+ present >15% of the information missing. In other words, less than 34/40 patients present a value of IFNγ, %perforin +, and %NKp46+, or %DNAM-1+ expression. [file Image_2.jpeg]

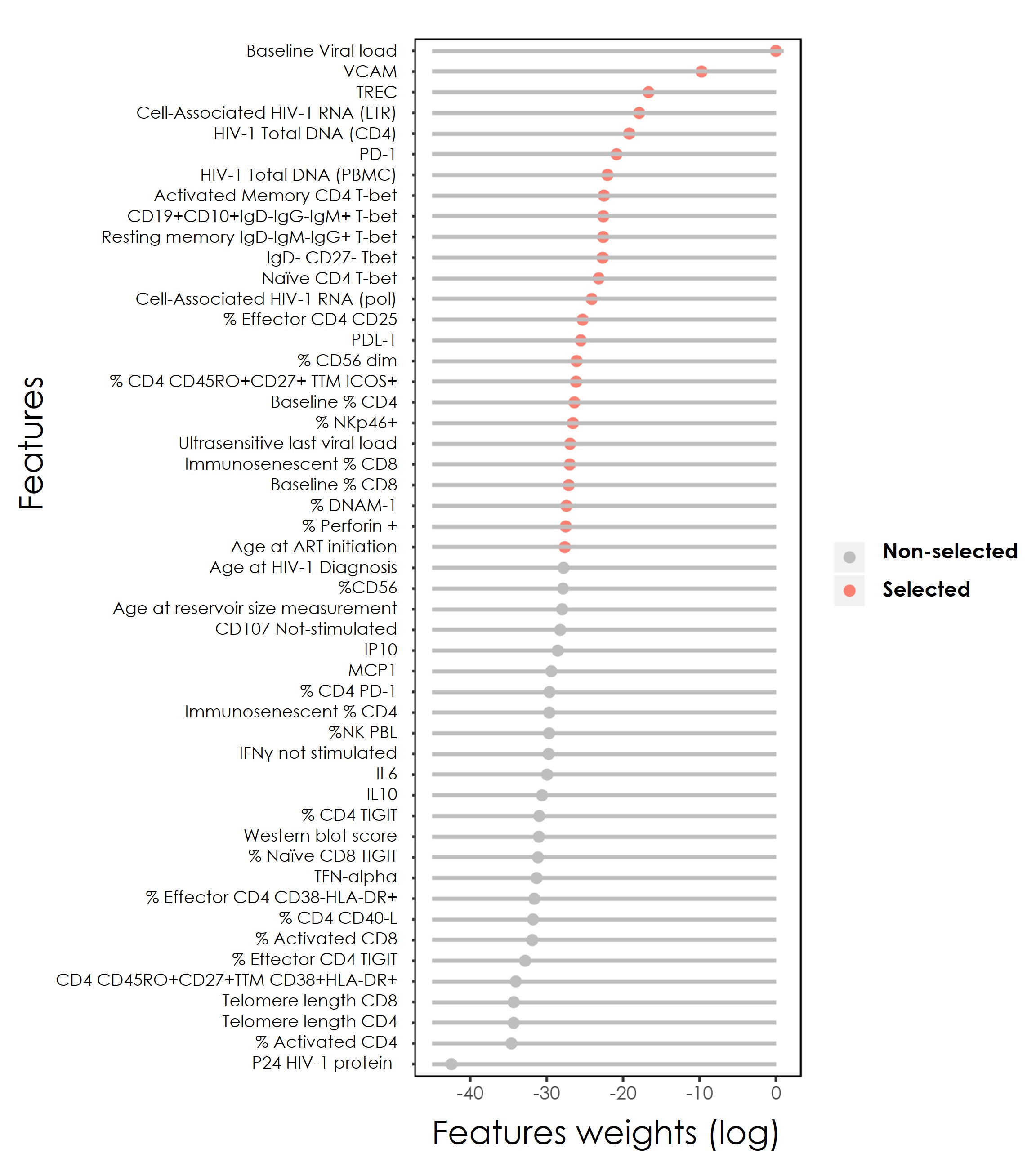

Supplement: Supplementary Figure 3 — Variable importance. Variable importance according to an unsupervised lasso-type penalty selection method for sparse clustering. We selected the most important features by a sequential forward search (SFS). A total of 25 variables with the highest feature weights were selected. [file Image_3.jpeg]
