# Supplementary material for: Clinical, Virological and Immunological Subphenotypes in a Cohort of Early Treated HIV-Infected Children
Source: Front Immunol. 2022 May 3;13:875692. doi: 10.3389/fimmu.2022.875692 (PMC9111748; doi:10.3389/fimmu.2022.875692)
Supplement: Supplementary Table 1 — Differences between complete cases database and after imputation. [file Table_1.docx]

**Table S1. Differences between complete cases database and after imputation**

|  | **Complete-cases** | **Imputed** | **p-value** |
| --- | --- | --- | --- |
|  | ***N=40*** | ***N=40*** |  |
| **Gender:** |  |  | 1.000 |
| Male | 13 (32.5%) | 13 (32.5%) |  |
| Female | 27 (67.5%) | 27 (67.5%) |  |
| **Age** |  |  |  |
| At HIV-1 diagnosis (months) | 4.17 [2.19;6.32] | 4.17 [2.19;6.32] | 1.000 |
| At ART (months) | 4.08 [0.25;6.23] | 4.08 [0.25;6.23] | 1.000 |
| At HIV-1 DNA reservoir measure (years) | 12.2 [8.03;15.6] | 12.2 [8.14;15.5] | 0.992 |
| **ART regimen at initiation:** |  |  | 1.000 |
| Triple NRTI | 2 (5.00%) | 2 (5.00%) |  |
| NNRTI | 1 (2.50%) | 1 (2.50%) |  |
| NRTI | 1 (2.50%) | 1 (2.50%) |  |
| NRTI + NNRTI | 23 (57.5%) | 23 (57.5%) |  |
| NRTI + PI | 12 (30.0%) | 12 (30.0%) |  |
| PI | 1 (2.50%) | 1 (2.50%) |  |
| **At Baseline** |  |  |  |
| HIV-1 RNA viral load | 409000 [95100;849940] | 409000 [95100;849940] | 1.000 |
| CD4 total count | 1515 [637;2235] | 1590 [721;2035] | 0.657 |
| % CD4 | 31.0 [18.0;38.0] | 32.8 [21.0;37.2] | 0.934 |
| % CD8 | 32.0 [25.0;40.0] | 31.9 [27.0;38.5] | 0.956 |
| **Time to suppression (months)** |  |  |  |
| Median [IQR] | 4.69 [2.52;6.26] | 4.69 [2.52;6.26] | 1.000 |
| **Anti-CMV IgG:** |  |  | 1.000 |
| Negative | 10 (25.6%) | 10 (25.0%) |  |
| Positive | 29 (74.4%) | 30 (75.0%) |  |
| **Anti-CMV IgM** |  |  | 0.973 |
| Negative | 38 (100%) | 40 (100%) |  |
| **Antigen/Antibody 4th generation Abbot:** |  |  | 1.000 |
| Equivocal | 2 (5.13%) | 2 (5.00%) |  |
| Non Reactive | 10 (25.6%) | 10 (25.0%) |  |
| Reactive | 27 (69.2%) | 28 (70.0%) |  |
| **Western blot** |  |  | 39 |
| Western blot score | 1.00 [0.50;2.00] | 1.19 [0.50;2.00] | 0.996 |
| HIV p24 | 0.00 [0.00;0.00] | 0.00 [0.00;0.00] | 0.967 |
| **Virologic features** |  |  |  |
| HIV DNA reservoir (copies /10^6^ PBMC) | 48.3 [6.65;113] | 48.3 [6.65;113] | 1.000 |
| HIV DNA reservoir (copies /10^6^ CD4) | 255 [75.0;434] | 290 [80.0;466] | 0.722 |
| HIV Cell-Associated RNA LTR (PBMC) | 2.73 [0.00;44.1] | 2.73 [0.00;44.1] | 1.000 |
| HIV Cell-Associated RNA pol (PBMC) | 0.00 [0.00;1.38] | 0.00 [0.00;1.38] | 1.000 |
| Ultrasensitive viral load | 2.37 [1.05;2.72] | 2.37 [1.05;2.72] | 1.000 |
| **NK cells** |  |  |  |
| %NK PBL | 5.03 [2.39;6.72] | 5.03 [2.39;6.43] | 1.000 |
| %CD56dim | 75.0 [68.8;81.5] | 74.4 [68.0;81.5] | 0.857 |
| %CD56- | 9.34 [5.57;16.7] | 9.98 [6.06;16.2] | 0.960 |
| %NKp46+ | 60.7 [49.9;74.0] | 60.8 [55.1;68.2] | 0.858 |
| %DNAM-1+ | 83.9 [77.2;89.4] | 83.0 [80.0;86.9] | 0.726 |
| CD107 Not-Stimulated | 6.41 [4.37;11.4] | 6.57 [4.66;10.9] | 0.738 |
| **Immunological profile** |  |  |  |
| % CD4 CD28– CD57+ immunosenescent cell | 1.63 [0.57;2.90] | 2.03 [0.74;3.14] | 0.698 |
| % IS CD8 CD28– CD57+ immunosenescent cell | 12.7 [8.01;16.8] | 12.9 [9.08;16.1] | 0.903 |
| % Activated cells CD4 ^+^ CD38+ HLADR+ | 0.37 [0.26;0.54] | 0.41 [0.28;0.54] | 0.746 |
| % Activated cells CD8 ^+^ CD38+ HLADR+ | 1.55 [1.00;2.00] | 1.73 [1.09;2.01] | 0.819 |
| Relative Telomere length CD4 | 1.33 [1.22;1.57] | 1.35 [1.23;1.56] | 0.917 |
| Relative Telomere length CD8 | 1.40 [1.25;1.52] | 1.39 [1.27;1.49] | 0.911 |
| TREC (PBMC) | 1720 [846;2730] | 1726 [877;2616] | 0.931 |
| % CD4 Effector CD38- HLA-DR+ | 1.73 [1.09;2.41] | 2.02 [1.23;2.39] | 0.786 |
| % CD4 Q2 CD45RO+ CD27+ TTM ICOS+ | 11.0 [7.56;17.2] | 11.1 [7.62;15.7] | 0.811 |
| % CD4 CD45RO+ CD27+ TTM Q10 CD38+ HLA-DR+ | 0.49 [0.30;0.66] | 0.48 [0.37;0.66] | 0.962 |
| % CD4 Effector CD25 | 24.6 [18.5;28.4] | 24.6 [19.2;28.0] | 1.000 |
| % CD4 Effector TIGIT | 1.62 [1.25;2.08] | 1.62 [1.29;2.06] | 0.934 |
| % CD4 TIGIT | 2.67 [2.26;3.63] | 2.93 [2.27;3.51] | 0.925 |
| % CD8 Naive TIGIT | 2.99 [2.29;3.74] | 3.01 [2.33;3.66] | 0.892 |
| % CD4 CD40L | 0.74 [0.44;1.98] | 1.08 [0.48;1.71] | 0.704 |
| % CD4 PD-1 | 4.12 [3.03;6.70] | 4.23 [3.08;6.12] | 0.909 |
| **B-cells T-bet expression** |  |  |  |
| Naïve | 188 [157;224] | 188 [157;224] | 1.000 |
| Activated Memory | 248 [204;325] | 248 [204;325] | 1.000 |
| Double Negative IgD- CD27- | 197 [143;237] | 197 [143;237] | 1.000 |
| Resting memory IgD- IgM- IgG+ | 186 [133;224] | 186 [133;224] | 1.000 |
| CD19+ CD10- IgD- IgG- IgM+ | 205 [154;256] | 205 [154;256] | 1.000 |
| **Cytokines** |  |  |  |
| PDL-1 (pg/mL) | 63.4 [53.4;76.4] | 63.4 [53.4;76.4] | 1.000 |
| IL-10 | 1.37 [1.03;1.66] | 1.37 [1.03;1.66] | 1.000 |
| IL-6 (pg/mL) | 0.88 [0.66;1.28] | 0.88 [0.66;1.28] | 1.000 |
| TNF-α (pg/mL) | 3.04 [2.33;3.74] | 3.04 [2.33;3.74] | 1.000 |
| PD-1 (pg/mL) | 389 [252;576] | 389 [252;576] | 1.000 |
| IP-10 (pg/mL) | 2.89 [1.98;4.64] | 2.89 [1.98;4.64] | 1.000 |
| MCP-1 (pg/mL) | 3.97 [2.64;4.82] | 3.96 [2.71;4.80] | 0.996 |
| VCAM (pg/mL) | 28842 [16731;42854] | 38788 [22607;45745] | 0.338 |
